# Following the legacy of professors Barbara Starfield and Leiyu Shi in Brazil as health policy: the National Health Survey (PNS), led by the Brazilian National Institute of Geography and Statistics (IBGE) and the Primary Care Assessment Tool (PCAT)

**DOI:** 10.1186/s12939-019-1083-2

**Published:** 2019-11-15

**Authors:** Erno Harzheim, Luiz Felipe Pinto, Otavio Pereira D’Avila, Lisiane Hauser

**Affiliations:** 1 Faculdade de Medicina, Universidade Federal do Rio Grande do Sul (UFRGS), Porto Alegre, Brazil; 2 Departamento de Medicina em Atenção Primária à Saúde, Faculdade de Medicina, Universidade Federal do Rio Janeiro (UFRJ), Rio de Janeiro, Rua Laura de Araújo, 36 – 2º andar – parte, Cidade Nova, Rio de Janeiro, CEP.21250-540 Brasil; 3 IHMT/Universidade Nova de Lisboa, Lisboa, Portugal; 4 Faculdade de Odontologia, Universidade Federal de Pelotas (UFPel), Pelotas, Brasil; 5 TelessaudeRS, UFRGS, Brazil, Rua Ramiro Barcelos, 2400, Santa Cecilia, Porto Alegre, RS CEP.90035-003 Brasil

**Keywords:** Primary health care, PCAT, Survey, Brazil

## Abstract

We present to the scientific community the pioneering of Brazilian National Institute of Geography and Statistics (IBGE, the Brazilian Census Bureau) in partnership with the Ministry of Health, the largest fieldwork ever conducted in a single country in the world, using the PCAT in a national household sample survey, visiting more than 100,000 households and 40% of the country’s municipalities. In Brazil, PCAT is being consolidated as an instrument to support public policy for the evaluation of primary health care. We believe that it represents a virtuous example of dialogue between scientific community and health management, following the legacy of Professors Barbara Starfield and Leiyu Shi.

Since the early 2000s, when the team led by professors Barbara Starfield and Leiyu Shi of the Johns Hopkins Bloomberg School of Public Health proposed to the scientific community the Primary Care Assessment Tools (PCAT) [1–3] to the evaluation of Primary Health Care (PHC) in the United States, dozens of other studies have been developed over the 20 last years on all continents, using validated versions adapted to the reality of each country, especially in North America, Latin America and most recently with the leadership of Professor Leiyu Shi in Asia [4, 5]. In low and middle-income countries, the access / first contact attribute remains the most difficult to reach when its PCAT score is calculated. Comprehensiveness is what most differentiates cities in each country in which the instrument has been used across continents [6].

But who could imagine that in almost 20 years later, a country of continental dimensions like Brazil, with huge social inequality, would adopt this instrument as an important part of a public policy, evaluating the Family Health Teams that work in primary health care and, to this end, would initiate the major household sample survey to create a national, regional and local baseline for each of the 27 federation units? Who would have thought that from Oiapoque to Chuí (from north to south of Brazil) the enormous challenge of collecting data with probabilistic household samples could be accomplished across the nation?

In Brazil, PCAT questionnaires using the same Starfield-Shi four-point Likert scale was validated and published in 2006 by Harzheim, Starfield, Rajmil, Álvarez-Dardet and Stein [7]. In 2010, Ministry of Health, with the technical support of Professor Starfield herself published PCAT expanded questionnaires [8]. To adapt it to the Brazilian reality, each original version of the instrument was transformed into an applicable tool by interviewers and went through a process of translation and reverse translation, adaptation, debriefing and validation of content and construct, as well as reliability analysis. From a scientific point of view, PCAT remains the one in which the techniques recommended by the Statistical Sciences for questionnaire validation are followed (multivariate / factorial analysis, correlation analysis, use of item-response theory, validity and reliability by Cronbach’s alpha coefficient) [9].

Until 2016, the largest sample in the world (*n* = 6675) performed in a single city occurred in Rio de Janeiro, Brazil, the second largest in the country, with the application of the instrument in expanded versions of adult and child users in family health units [10], including the most known poor community of Brazil, the neighborhood of Rocinha [11]. In Spain, the Catalonia region included a reduced PCAT version for both adult and children [12] in a national survey in 2006, with the participation of Professor Barbara Starfield.

Like the United States Census Bureau, Brazilian National Institute of Geography and Statistics (IBGE) is huge, and has a national structure with more than 500 support offices in all five regions of the country. However, the giant heterogeneity of each federation unity and the difficulty of connectivity and linkage of administrative databases do not allow the use of interviews in national surveys by telephone or email. Therefore, a true “war operation” was planned in detail for the five-year National Health Survey (PNS), the main sample household survey of the country that portrays the most diverse facets of Brazilian population.

PNS emerged from breaking down the major growth of elements included in the National Household Sample Survey (PNAD) Special Health Supplements, published every 5 years since 1998 (1998, 2003, 2008, and then for the first time in 2013 as PNS), maintaining the same research aspects (set of questions or Modules) so that data may be compared to previous surveys. This survey is part of the IBGE Integrated System of Household Surveys (SIPD), and uses the “Master Sample” infrastructure. Sampling for PNS-2019 was designed to produce indicators for 80 geographic sections: the country, five regions, 27 states, 21 metropolitan regions and 27 state capitals (this one the maximum level of granularity allowed). It is important to point out the need to disclose estimates and their coefficients of variation.

Encouraged by one of the authors of this text, in August 2019, IBGE in partnership with the Ministry of Health started the survey fieldwork visiting more than 100,000 households in about 2000 municipalities (about 40% of the country’s municipalities) from all over the 27 states and regions of the country, interviewing an adult aged 18 or older in each household, and applying for the first time in its 20 years of Health Surveys, the reduced validated version of the PCAT, as a part of one of the PNS set of questions (Module H) [13].

To the best of our knowledge, it will be the first time that a country in the world applies the instrument on a nationwide sample scale, albeit in a reduced version so that it can draw a baseline for further comparisons in future national surveys.

Following the legacy of Professors Barbara Starfield and Leiyu Shi, we believe that strong primary care should follow the essential and derivative attributes stated in the 1990s by Shi and Starfield, who later had her work translated into Portuguese by United Nations Educational, Scientific, and Cultural Organization (UNESCO) and launched in Brazil in the same year 2002 [14]. With Brazil’s pioneering health household national experience in 2019, PCAT is being consolidated as an instrument to support public policy for the evaluation of primary health care. It also considers a new federal financial model of pay for performance that will begin in 2020 [15]. This match between scientific community and public health policy therefore represents a virtuous example of success in this partnership. So our tribute to Professors Barbara Starfield and Leiyu Shi is a way to keep the flame burning and the legacy built and under construction to demonstrate that yes, a nationwide scientific survey and primary care services integration is possible with representative random samples, from a statistical point of view, that is, with external validity, and its results can be generalized to the population as a whole, disclosing the scores found and their respective coefficients of variation.

## Data Availability

Not applicable. The National Survey of Health (PNS-2019) has just started to collect data from 100,000 household all over the 27 States of Brazil. The authors chose the section “Letter to the Editor” modality, since the objective is to inform readers of what is considered as something unprecedented in the use of the PCAT instrument, that is, they believe it is the first time that a continental country such as Brazil conducted a national household sample survey with the application of the this evaluation tool in all regions, federation units and capitals of a country.
